# Integration of metabolomics, lipidomics and clinical data using a machine learning method

**DOI:** 10.1186/s12859-016-1292-2

**Published:** 2016-11-22

**Authors:** Animesh Acharjee, Zsuzsanna Ament, James A. West, Elizabeth Stanley, Julian L. Griffin

**Affiliations:** 1 Medical Research Council, Elsie Widdowson Laboratory, 120 Fulbourn Road, Cambridge, CB1 9NL UK; 2 The Department of Biochemistry and Cambridge Systems Biology Centre, University of Cambridge, 80 Tennis Court Road, Cambridge, CB2 1GA UK

## Abstract

**Background:**

The recent pandemic of obesity and the metabolic syndrome (MetS) has led to the realisation that new drug targets are needed to either reduce obesity or the subsequent pathophysiological consequences associated with excess weight gain. Certain nuclear hormone receptors (NRs) play a pivotal role in lipid and carbohydrate metabolism and have been highlighted as potential treatments for obesity. This realisation started a search for NR agonists in order to understand and successfully treat MetS and associated conditions such as insulin resistance, dyslipidaemia, hypertension, hypertriglyceridemia, obesity and cardiovascular disease. The most studied NRs for treating metabolic diseases are the peroxisome proliferator-activated receptors (PPARs), PPAR-α, PPAR-γ, and PPAR-δ. However, prolonged PPAR treatment in animal models has led to adverse side effects including increased risk of a number of cancers, but how these receptors change metabolism long term in terms of pathology, despite many beneficial effects shorter term, is not fully understood. In the current study, changes in male Sprague Dawley rat liver caused by dietary treatment with a PPAR-pan (PPAR-α, −γ, and –δ) agonist were profiled by classical toxicology (clinical chemistry) and high throughput metabolomics and lipidomics approaches using mass spectrometry.

**Results:**

In order to integrate an extensive set of nine different multivariate metabolic and lipidomics datasets with classical toxicological parameters we developed a hypotheses free, data driven machine learning approach. From the data analysis, we examined how the nine datasets were able to model dose and clinical chemistry results, with the different datasets having very different information content.

**Conclusions:**

We found lipidomics (Direct Infusion-Mass Spectrometry) data the most predictive for different dose responses. In addition, associations with the metabolic and lipidomic data with aspartate amino transaminase (AST), a hepatic leakage enzyme to assess organ damage, and albumin, indicative of altered liver synthetic function, were established. Furthermore, by establishing correlations and network connections between eicosanoids, phospholipids and triacylglycerols, we provide evidence that these lipids function as a key link between inflammatory processes and intermediary metabolism.

**Electronic supplementary material:**

The online version of this article (doi:10.1186/s12859-016-1292-2) contains supplementary material, which is available to authorized users.

## Background

The metabolic syndrome (MetS) and its associated conditions such as insulin resistance, dyslipidaemia, hypertension, hypertriglyceridemia, and obesity are all considered global health problems, and contribute to cardiovascular disease and increased mortality and morbidity [1]. Beneficial effects for the treatment of diabetes and MetS by peroxisome proliferator-activated receptors (PPARs) are well established [2]. However, considerable controversy remains about their general safety and side effects in the liver, the cardiovascular system and skeletal muscle. The search for new, and less toxic agonists are of prime importance and several new strategies are being explored to overcome undesirable treatment effects, such as increased risks associated with certain cancers when administered long term in animal models. One such strategy has been the simultaneous activation of two or three (−pan) PPAR receptors in order to favourably influence pathways associated with MetS, while negating some of the side effects such as increased adiposity caused by PPAR-γ agonists. Compound development, where inhibition or activation of enzymes beyond what would be considered the primary PPAR targets are also being explored, including PPAR-pan treatment alongside Sirtuin (SIRT 1) activation [3], or the use of PPAR-pan activators in conjunction with cyclooxygenase (COX) inhibition [4]. The development of better delivery systems such as the use of nano-capsules are also being explored [5].

In the current study, the effects of a PPAR-pan agonist on liver metabolism was investigated after dietary treatment of male Sprague–Dawley (SD) rats. Classical toxicological tests (clinical chemistry) and mass spectrometry (MS) approaches for metabolomic and lipidomic [6] changes were used to provide a ‘deep phenotype’ for the animals. High-throughput ‘-omic’ technologies have gained much interest in recent years and have been previously employed in order to unravel disease mechanisms associated with MetS [7–10].

Despite technological advancement within metabolomics, there are still limitations with the approach. Not only does the diverse and structurally complexity of many metabolomes remain a challenge, the understanding, interpretation and integration of large datasets in conjunction with classical toxicological parameters is a major task. An integrative approach is needed in order to understand the principles underlying the metabolic regulation of a system and how their combined interactions associates with variation in clinical phenotypes results in pathophysiology. This challenge demands new data exploration strategies such as analysis workflows, statistical and computational algorithms for data integration, filtering, and network analysis if we are truly going to be able to convert the large multivariate data collected during such metabolomic experiments into new biological knowledge.

Here we applied a machine learning approach called Random Forest (RF) [11], which is able to integrate multiple data types and successfully combine classical clinical chemistry and toxicology test results with multivariate metabolomic and lipidomic data. Several authors have previously adapted RF methods for data integration (also referred to as data fusion) including Acharjee et al. who applied a RF approach to integrate transcriptomics and metabolomics data in plant spices [12], and Fortino et al., who developed and evaluated a fuzzy logic combination with RF to prioritize the candidate discriminant features from gene expression data [13]. Briefly, a RF is a collection of decision trees (ensembles) where each tree gets a “vote” in classifying the sample and find patterns in the data. While RF approaches are very powerful for multivariate datasets, currently they are scarcely applied in metabolomic studies [14]. In the present study, RF classification was used to select subsets of metabolites from a ‘real life’ metabolomics study showing that this statistical approach is successful in building associations and predicting different doses in a drug safety assessment environment. Next, an RF regression approach was used to link liver metabolites with classical clinical chemistry parameters measured from plasma. An integrated network analysis was performed providing a relatively small set of interrelated metabolites which were predictive of dose levels, whilst also aiding understanding of the metabolic processes involved in the study. Metabolites selected in this way provide a useful starting point to understand the underlying effects of PPAR-pan treatment and also aided the generation of hypotheses to be further investigated using more targeted analyses. In addition to exploring the selected metabolites and their known relationships to the PPAR system, one of the emerging hypotheses from our analyses, the central role of eicosanoids (and other oxygenated metabolites of polyunsaturated fatty acids) following PPAR-pan action, were followed up by using an additional MS method for the detection of eicosanoids in the subset of the samples biologically validating our approach. For the purposes of this article we have used the term lipid mediators to encompass both classical eicosanoids and oxidized lipids that are shorter and longer than arachidonic acid.

## Methods

All the datasets and protocols used for generating them are based on the Ament et al., [15] manuscript where we have previously published the study design and some of the metabolomics data. These are briefly outlined below, along with the description of the RF data fusion approach used in the present study.

### Chemicals

Aqueous metabolite standards were purchased form Sigma Aldrich (Poole, Dorset, UK). Lipid standards were purchased form Avanti-Polar lipids (Alabaster, Alabama, US) with the exception of the standard mix containing 37 fatty acid methyl esters (FAMEs) (Sigma Aldrich, Poole, Dorset, UK). Deuterated compounds used as internal standards were purchased from Cambridge Isotope Laboratories (Andover, Massachusetts, US). Solvents used were of HPLC grade or higher. All other compounds, chemicals and solutions used are detailed where relevant.

### Animal experiments and study design

All animal studies were ethically reviewed and carried out in accordance with the Animals (Scientific Procedures) Act 1986 and the GSK Policy on the Care, Welfare and Treatment of Animals.

**Fig. 1:**
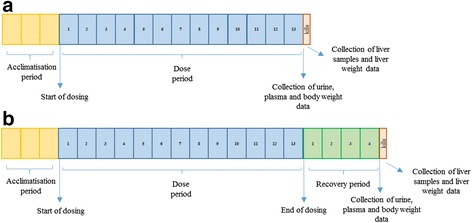
The design of the PPAR-pan agonist treatment study. **a** Four week old Sprague Dawley rats were acclimatised for approximately 3 weeks and were killed after a 13 week dose period. **b** Recovery animals were acclimatised for 3 weeks, dosed with the test compound for 13 weeks and kept for an additional 4 week dose free period. Before terminal kills urine and plasma samples were collected for urinalysis and clinical chemistry. After the terminal kills liver weight were recorded and liver samples were collected for metabolomic analysis

The PPAR-pan activator was administered to male Sprague–Dawley rats (Crl:CD (SD) strain), 12 animals per group, by daily oral gavage at 30,100, 300, 1000 mg/kg/day for 13 weeks. A separate satellite group of animals (6 per group) were kept for a 4 week treatment free period in the control, intermediate 2 (300 mg/kg/day) and high (1000 mg/kg/day) dose groups. The detailed number of animals used and doses are shown in Table 1. Rats of the Crl:CD (SD) strain were obtained from Charles River UK Ltd to provide 78 healthy animals which were randomly allocated to study groups. They were acclimatised for approximately 3 weeks and a veterinary inspection was performed before the start of dosing. On Day one of treatment, the rats were approximately 7 weeks old. The study design is summarised in Fig. 1a and 1b. The number of the recovery animals were decided in accordance with the principles of the 3Rs (replacement, reduction and refinement) and only six animals were used in each group in order to minimise the number of animals killed.

**Table 1:** Animal identifiers and study design

Group Description	Dose (mg/kg/day)	Animal number	Recovery animals
Control	0	1–12	13–18
Low	30	19–30	-
Intermediate 1	100	31–42	-
Intermediate 2	300	43–54	55–60
High	1000	61–72	73–78

### Plasma samples for clinical chemistry analysis

Blood samples were taken from the lateral caudal vein using lithium heparin anticoagulant at weeks 4 and 13. Samples were mixed gently and placed on wet-ice until centrifugation. The resultant plasma was separated and 180 μL of each was transferred to vials containing 9 μL of 25 % glacial acetic acid in water. Samples were mixed thoroughly, immediately frozen and stored at −80 °C.

In total 34 parameters were measured, among which were aspartate aminotransferase, alkaline phosphatase, potassium, inorganic phosphorus, total protein, total cholesterol, urea, and alanine aminotransferase. Further, relative liver weight was measured which is the ratio between the measured body weight and the measured liver weight of each animal. A complete list of clinical chemistry and their full descriptions can be found in Additional file 1.

### Tissue samples

Tissue samples were collected following an overdose of anaesthetic (halothane Ph. Eur. Vapour). Samples of the liver were immediately removed, weighed, and sections snap-frozen in liquid nitrogen. Samples were maintained at −80 °C until further analysis.

An array of analytical methods were used to examine the metabolomic and lipidomic profile of tissues, including gas-chromatography mass-spectrometry (GC-MS), direct infusion mass spectrometry (DI-MS) and liquid chromatography tandem mass spectrometry (LC-MS/MS). Ten datasets were generated comprising of hepatic total fatty acids by GC-MS, intact lipids by DI-MS (positive and negative mode), intact lipids by LC-MS/MS(positive and negative mode), acyl-carnitines, eicosanoids and targeted aqueous metabolites, aqueous open profile (positive and negative mode), comprising approximately 1000 variables.

### Liquid-liquid extraction procedures from liver samples

Methanol: chloroform solution (2:1, 600 μL) along with a stainless steel ball (Qiagen, Hilden, Germany) was added to approximately 50 mg of frozen tissue and homogenised with a Tissue Lyser (Qiagen, Hilden, Germany). Chloroform and water (200 μL each) were added, samples were sonicated for 15 min and centrifuged (13 500 rpm, 20 min). The resulting aqueous and organic layers were separated and the extraction procedure was repeated.

The resulting aqueous and organic layers were separated and the extraction procedure was repeated. Samples were dried under nitrogen before processing for GC-MS and LC-MS. GC-MS, and LC-MS/MS methods for lipid extraction and analysis were carried out according to methods previously described [16, 17]. Methods published in previous publications are also provided as supplementary for convenience (Additional file 2). Those methods that are unpublished are detailed below.

### Analysis of intact lipids by direct infusion mass spectrometry (DI-MS)

An aliquot of 30 μL of the organic stock solution was added to 20 μL of internal standard mix in methanol: chloroform (1:1). The internal mix represents different classes of lipids that are used to check mass accuracy to aid identification of lipids species (2.5 μM 1,2-di-o-octadecyl-sn-glycero-3-phosphocholine, 5 μM 1,2-di-o-Phytanyl-sn-glycero-3-phosphoethanolamine, 2.5 μM C8-ceramide, 2.5 μM N-heptadecanoyl-D-erythro-phingosylphosporylcholine 25 μM undecanoic acid, 2.5 μM trilaurin and 5 μM β-sitosterol acetate). Of the resulting solution, 20 μL was transferred to a glass coated 96 well plate each containing 80 μL 15 mM ammonium acetate in 2:1 isopropanol: methanol. The instrumentation comprised of an Exactive Orbitrap Mass Spectrometer (Thermo Scientific, Hemel Hempstead, Hertfordshire, UK) coupled to a robotic nanoflow TriVersa Nanomate ion source (Advion Biosciences, Ithaca, NY, US) using nanoelectrospray chips with a spraying nozzle diameter of 4.1 μm.

Mass spectrometry data was collected for 1 min in positive ionisation (+1.5 kV) mode followed by 1 min in negative ionisation (−1.5 kV) mode. The ion transfer capillary was set at a temperature of 250 °C for negative ionisation mode and 225 °C for positive ionisation mode. For every ten samples one blank and one pooled sample were added to ensure quality control.

### LC-MS/MS analysis for the open (non-targeted) profiling of aqueous metabolites

For LC-MS/MS analysis of small molecules, aqueous phase metabolites resulting from the chloroform-methanol extraction were used. The entire fraction was dissolved in 300 μl of 70:30 acetonitrile: water containing 20 μM universally ^13^C- and ^15^N- labelled glutamate and 20 μM universally labelled succinate. Samples were vortex mixed, sonicated, centrifuged (17,000 × g, 5 min) and pipetted into auto sampler vials. Chromatographic analyses were performed using an Acquity Ultra Performance Liquid Chromatography (UPLC) system with an Acquity UPLC BEH amide 1.7 μm column (2.1 × 100 mm) coupled to a Xevo-G2 Quadrupole Time-of-Flight (Q-ToF) with a Z-spray electrospray source (Waters Corporation, Elstree, Hertfordshire).

The mobile phase gradient was run at 0.6 mL/min using mobile phase A containing 0.05 % ammonium hydroxide in 10 mM ammonium acetate; mobile phase B was acetonitrile. The initial mobile phase comprised 10 % A. This was subsequently increased to 50 % A over 7 min, after which the gradient was returned to 10 % A for 3 min. Data were acquired in both positive and negative ionisation modes using a source temperature of 150 °C and a desolvation temperature of 300 °C. The desolvation gas flow was 700 L/h and the acquisition mass range was 50 – 1500 m/z.

For automated peak-picking MarkerLynx™ within the software suite MassLynx™ (version 4.1) by Waters Ltd. (Elstree, Hertfordshire, UK) was used. Mass to charge ratio and intensity pairs were normalised to the internal standard glutamine [U- ^13^C, ^15^N] in positive ionisation mode and to universally labelled ^13^C-succinate in negative ionisation mode. For identification, exact mass data were searched against the metabolite mass spectral database METLIN (http://metlin.scripps.edu/metabo_advanced.php).

**Fig. 2:**
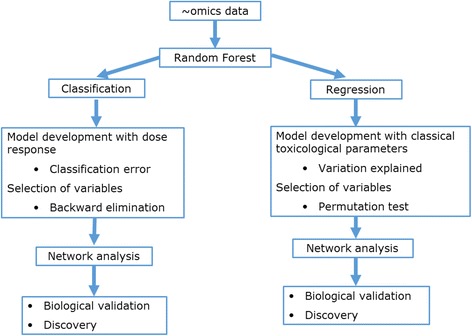
Methodological work flow for data integration. Random forest (RF) classification was used to select subsets of metabolites from the combination of all metabolite data sets. Data sets which are the best in predicting the dose of PPAR-pan administered were assessed by calculating classification error values. The variables from the individual datasets were selected by a backward elimination approach, and the final set of metabolites were used for network analysis. As a separate strategic workflow, an RF regression approach was used to link liver metabolites with classical clinical chemistry parameters. Datasets which explain the variation of the classical clinical chemistry parameters were calculated, and individual variables were selected using permutation tests. Again, the final set of metabolites and the explained clinical chemistry parameters were selected for network analysis

The final data set from this analysis was autoscaled for further analysis. Autoscaled variables have a mean of zero and a variance (and also standard deviation) of one, thereby giving all variables (metabolites) an equal weight in the analysis. The analysis methodology is shown in Fig. 2.

### Availability of data

List of lipidomics, metabolomics and clinical chemistry data set are provided in the Additional file 3.

### Random forest

We used Random Forest (RF) for data fusion which is a machine learning ensemble method in conjunction with multiple learning algorithms to obtain better predictive performance [11]. The major difference between machine learning and linear methods is that, linear methods assume all metabolites are combined in a linear way and then impact on the outcome, for example control compared with high dose, whereas RF does not assume any linearity and rather uses the sum of piecewise functions, and hence is able to discover more complex dependencies. RF can be used for both classification and regression [11] based analysis on the data we have generated in this study. For example using liver metabolites (GC-MS, LC-MS/MS, DI-MS) and plasma clinical chemistry datasets to classify the different dose groups and also cross-correlating between the different datasets. Random Forest (RF) uses a bootstrapping method for training or testing and decision trees for prediction. The bootstrapping process generates random samples from the dataset with replacement. Every bootstrapped sample has a corresponding left out or ‘out-of-bag’ (OOB) sample which is used to test the routine, and prediction is made on those left out samples.

#### Random forest classification

We used RF as a multiclass classification method using the control group, four different doses, and the three recovery groups as different classes, whilst metabolomic and lipidomic mass spectrometry data (acyl carnitines, aqueous metabolites, DI-MS, GC-MS, intact lipids) were treated as the predictor sets separately.

RF needs to use the number of trees (ntree) and number of variables (metabolites) randomly sampled as candidates at each split (mtry), and these parameters need to be defined. We used ntree = 500 and mtry = square root of variables in our models. For example, for the acyl carnitines data set, the mtry value was set to the nearest integer to the square root of 40 which is 6. Choosing the parameters was done based on the method described by Liaw and Wiener, 2002 [18].

#### Random forest regression

RF was used for regression of the classical clinical chemistry phenotypic traits with the metabolomic and lipidomic mass spectrometry data (acyl carnitines, aqueous metabolites, DI-MS, GC-MS, and intact lipids) being treated as predictor sets separately. RF constructs a predictive model for each of the response clinical phenotypes, whilst qualifying the importance of each variable. In this case the aqueous and organic metabolites, and their ability to explain the variation present in the classical clinical chemistry phenotypes. We quantified the RF regression model based on the variation explained by the model. The variation explained by RF is not just a measure of the goodness of fit of the data, but is also determined by the left-out samples (the “out-of-bag” samples), so it should be interpreted as a measure of predictive quality (here considered as the Q^2^). The variance explained in RF is defined as:

1-(Mean square error (MSE)/Variance of response), where MSE is the sum of squared residuals of the OOB samples divided by the OOB sample size. Since the MSE is estimated on the OOB samples and the total variance on all the samples, Q^2^ can be negative. RF regression also needs to use some of the parameters used above in the classification models; for example number of trees (ntree), and number of variables (metabolites) randomly sampled as candidates at each split (mtry). We used ntree = 500 and mtry = one third of the variables. For example, for the acyl carnitines data set, the mtry value will be one third of 40 which is approximately 13. Choice of these parameters was done based on Liaw and Wiener, 2002 [18].

#### The Backward elimination method

To select metabolites we iteratively fitted random forests, at each iteration building a new forest after discarding 20 % of the metabolites with the smallest variable importance. The selected set of metabolites is used as a predictor to fit the model to check the OOB error rate. This procedure is done iteratively using the varSelRF function from the varSelRF package in R [19].

#### Permutation test

RF quantifies the importance of metabolites that explain the variation present in the clinical phenotypes, but does not give a significance level or a threshold to choose a possible subset of associated metabolites. Therefore, we included a permutation test to indicate significance of the metabolite association in this study. In our situation, we randomized all clinical phenotypes separately and each time applied RF. The RF model was applied 1000 times for 1000 different randomizations of the clinical phenotypes and in each analysis we estimated the variance explained by the RF model (Q^2^) and variable importance of all variables in terms of a decrease in node impurities. We ordered node purity values from the permuted data sets and took the 95 percentile from the distribution impurity values for node impurity to assess the significance of the of individual genes and metabolites. The same was done for Q^2^ values of the model: the 95-percentile was used as a cut-off to denote significance of the Q^2^ value in RF regression.

For classification purposes, we used backward selection and for regression, we used a permutation test for assessing the significance of the metabolites.

#### Network analysis

A network is a set of nodes and a set of edges, where each node represents either a metabolite or classical clinical chemistry parameter, whereas the edges represent associations amongst them. A partial Pearson correlation coefficients were used to quantify the strength of association between combinations of metabolites or clinical chemistry parameters. A significance threshold of α = 0.05 was used to draw edges between the selected nodes. We used the partial correlation because it measures the correlation between two variables after their linear dependence on other variables is removed. It can distinguish between direct and indirect associations whereas correlation-based network cannot and often yield many spurious edges [20]. Partial correlation analysis was done using the ppcor package in R [21].

### Software

All statistical and network analysis was done in using R software (v3.2.1). We used two R packages for Random Forest analysis: randomForest and varSelRF. The R- Scripts are provided in the Additional file 4.

## Result and discussion

### Random forest (RF) classification identifies the most informative mass spectrometry platforms for determining dose response effects

**Fig. 3:**
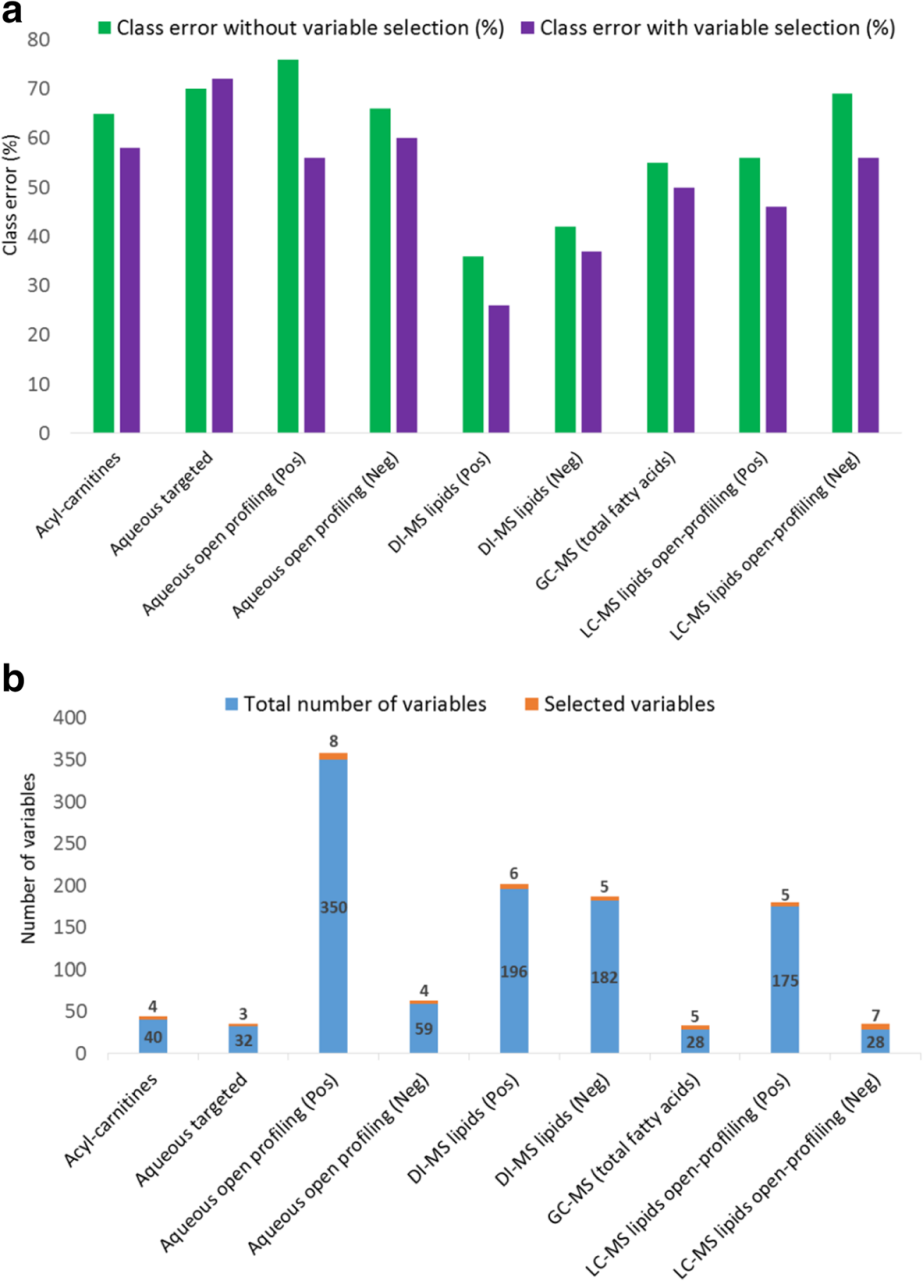
Random Forest (RF) classification approach for the determination of class error (how well the PPAR-pan dose level is predicted) and the selection of variables (which variables contribute to PPAR-pan dose level prediction) in each different dataset. **a** Class error of metabolomic and lipidomic dataset comparing values using the full set of variables and selected variables for calculations. **b** The number of variables contained within each dataset (in blue) and the number of metabolites after variable selection (in orange). For example, the number of total acyl-carnitines is 40 (in blue) and only four were selected (in orange)

Using the RF classification approach, four different doses in addition to the control and the three recovery groups were treated as multiclass parameters, whilst metabolomic and lipidomic mass spectrometry data were treated as predictor sets. OOB misclassification error rates were calculated for the individual datasets. DI-MS in positive ionisation mode, measuring primarily glycerophospholipids and glycerolipids, was found to have the lowest OOB errors of 36 % and hence the most informative compared to other datasets, whilst aqueous open profiling (positive mode) metabolite analysis data resulted in the highest class error of 72 % and was therefore the least predictive of dose levels administered (Fig. 3a). Across the different metabolite datasets the most discriminatory metabolites were selected using a backward elimination approach (Fig. 3b) [19]. The number of selected metabolites, were surprisingly small, making the data matrix considerably smaller and most importantly, simplifying the biological information needed to be considered. The selected subset of metabolites were used to re-calculate the OBB misclassification errors and it was found that the class error improved in eight out of nine datasets using these reduced datasets (Fig. 3a). Variable selection using RF classification is a rapid, and an effective approach that allows one to comprehend the complexity of the data matrix. (relative concentration changes of the selected variables are displayed using box plots and are provided in Additional file 5). The selected metabolites from the RF analysis (56 in total) were combined, and re-subjected to the RF classification approach. The OOB error rates improved to 22 % (from 36 % for the best dataset considered on its own previously); whilst re-applying a backward elimination process the most discriminatory variables reduced to 12 and lowered the OOB error even further to 21 %.

### Partial correlation network analysis as an effective tool for visualising metabolic pathways

**Fig. 4:**
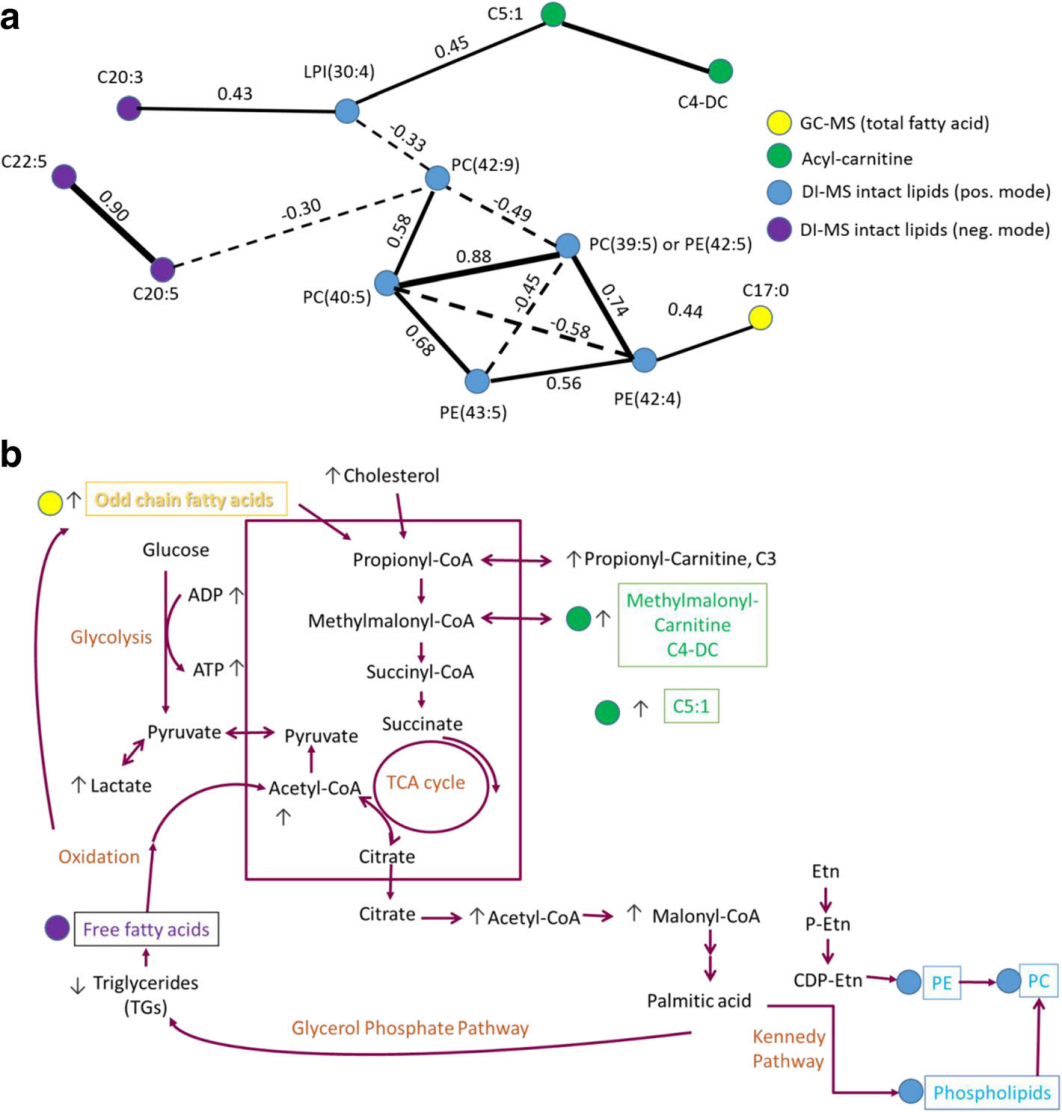
Network of selected metabolites. **a** A partial correlation network of the most discriminatory metabolites (12) differentiating between different doses of the PPAR-pan treatment concentrations. The solid lines denote positive and dotted lines denote negative correlations, and the thickness of the lines indicate the strength of the associations. **b** Biological pathways and their potential connections associated with the selected 12 metabolites. Note that metabolites of interest that were detected by our RF approach are color-coded on both pathway maps

As a next data processing step, the partial correlation between the selected 12 metabolites were calculated. The generated network figure (Fig. 4) provides an interpretable and useful view of metabolite connections.

Four broad themes emerged from the analysis. Firstly, the selected 12 metabolites include only lipids, and no aqueous compounds, reflecting the intimate role of PPARs in lipidomic remodelling. Secondly, changes in acyl-carnitines (C4-DC and C5:1) are suggestive of potential aciduria [22]. The building up in aciduria comes from increased glycolysis and production of lactate as a result of impaired mitochondria. As PPARs are known to regulate mitochondrial and peroxisomal lipid metabolism, and aciduria is commonly reported in mitochondrial disorders, this suggests common pathophysiological mechanism of damage. Thirdly, we noted that metabolites C20:3, C20:5, C22:5 are all precursors of eicosanoids or lipid mediators that can act as signalling molecules. Furthermore, all phospholipids highlighted by our RF approach contain at least four double bonds, and hence these species also potentially feed into the arachidonic acid cascade and eicosanoid production. Changes in PCs with at least four double bonds, most likely represent lipid remodelling to generate eicosanoid species derived from arachidonic acid.

Finally, the odd chain saturated fatty acid: C17:0, commonly considered to be a marker of ruminant fat intake [23], was also found as an important dose predictor and was highly discriminatory, leading us to further speculate on suggestions linking this fatty acid to fatty acid α-oxidation [23], possibly in peroxisomes.

### The RF regression approach for understanding toxicology in the era of -omics technologies

Thorough understanding of new methods is essential in order to avoid misinterpretation of data which could lead to false conclusions about a complex biological process like toxicity. Data generation for new biomarkers that are used to characterise and describe cellular responses are growing exponentially in the post genomic era [24]. This information has an unrealised power to provide increased understanding on toxicological outcomes. While the potential of -omic approaches have been realised by regulatory agencies for improving the risk assessment process, the strategy for evaluating cell and tissue damage from a toxic insult has changed very little for almost sixty years [25, 26].

**Fig. 5:**
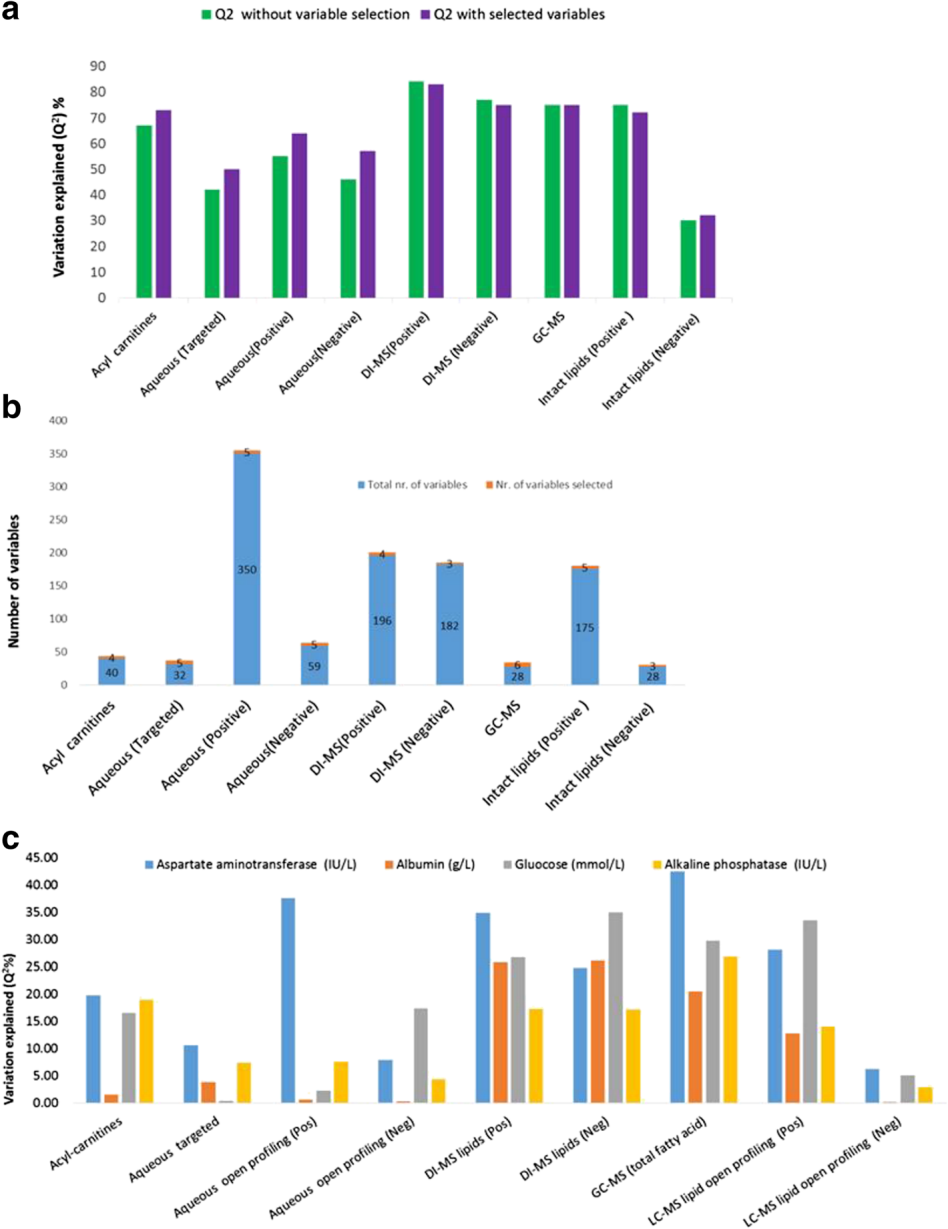
Variations explained by metabolomic and lipidomic datasets in relative liver weight and clinical chemistry parameters using the random forest (RF) regression approach. **a** Variation explained (Q^2^) in relative liver weight with and without variable selection. **b** The number of selected variables compared to the original (full dataset) number of variables. **c** Variation explained (Q^2^) with all the metabolomic and lipidomic data linking with phenotypes associated with plasma clinical chemistry from liver analysis. In total four parameters showed the largest variation explained across the different data sets

In an attempt to merge metabolomics and lipidomics data with different clinical chemistry phenotypes, such as relative liver weight and plasma clinical chemistry parameters, an RF regression approach was used. First, relative liver weight was examined, and the variation explained by metabolomic and lipidomic datasets in relative liver weight was calculated. Metabolites explaining the variation were filtered out form the rest of the data, and the RF regression calculations were repeated. There were no significant differences in the variations explained (Q^2^) when comparing full datasets to only selected metabolites (Fig. 5a), although the number of metabolites driving the variation were much smaller when compared to the full dataset (Fig. 5b). The intact lipids measured in positive ionisation mode by DI-MS were found to explain the highest variation (84 %) with regards to relative liver weight changes and the lowest variation was explained by intact lipid LC-MS data in negative ionisation mode (32 %).

**Fig. 6:**
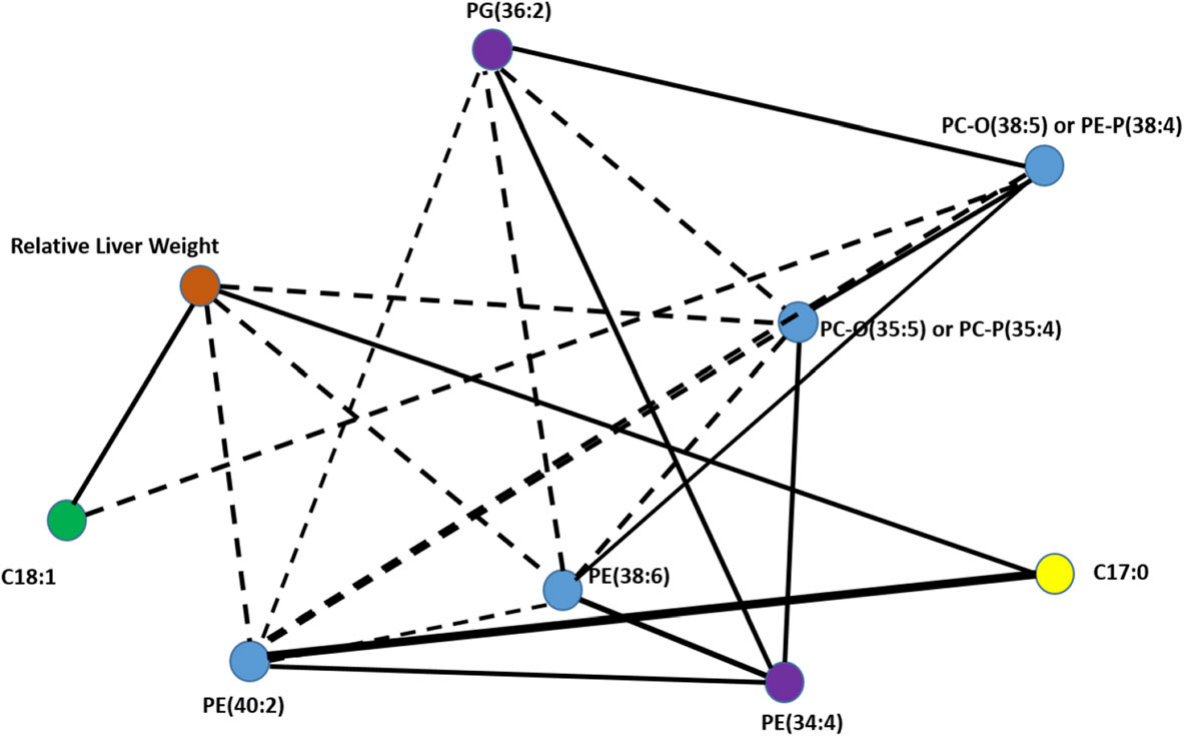
A partial correlation network of the nine selected variables linking with the relative liver weight (ratio between the measured body weight and the measured liver weight of each animal). Different types of data are shown in different colours. The thickness of the lines relate to the extent of the correlation, where straight lines indicate positive and dotted lines indicate negative correlations

The plasma clinical chemistry parameters were investigated in a similar fashion to the relative liver weight, with the exception, that this dataset contains multiple (34) clinical chemistry variables as outcomes, each of which were examined, and the metabolites explaining their variation identified. The highest values for variations explained include aspartate aminotransferase (AST), albumin, glucose and alkaline phosphatase (ALP) resulting in 42, 26, 35 and 27 % variation explained (Q^2^), respectively (Fig. 5c). It is also apparent form the data, that GC-MS of total fatty acids and DI-MS measurement of lipids measured in both positive and negative ionisation modes performed the best in explaining the variation in the parameters (Fig. 5c). A partial correlation network diagram with relative liver weight is shown in Fig. 6. Information of the other 30 variables assessed is provided in Additional file 6

.

The phospholipids explaining the highest variation in relative liver weight are highly unsaturated, and therefore, structurally similar to the lipids identified in the dose response analysis described above. It is likely that these species can feed into the eicosanoid cascade of arachidonic acid metabolism. There are two well established degradation pathways of highly unsaturated fatty acids, one is the *β*-oxidation pathway in mitochondria and peroxisomes and the other is the formation of eicosanoids. PPARs play a key role in both metabolic processes. However, we also note, again, an unexpected change in the C17:0 total fatty acid content, which might link the PPAR system to a thus far under-investigated possibility of fatty acid α-oxidation pathway.

### Investigation of eicosanoid production from intact lipids: Biological perspective

In order to validate the changes in lipid mediator precursor fatty acids C20:3, C20:5, C22:5 and their role in predicting dose response, and to investigate the possibility that the highly unsaturated phospholipids feed into the arachidonic acid cascade, an additional dataset was generated focusing on the measurement of eicosanoids. Since dose responses were highly predictable, only a subset of the study samples were analysed, including the control, the intermediate dose 2 (300 mg/kg/day) and the highest dose group (1000 mg/kg/day) samples. Eicosanoids are known to possess pro- or anti- inflammatory properties, and the liver is highly responsive to inflammation as it is densely populated with its own macrophages, the Kupffer cells, which account for over 10 % of total liver cells [27]. Importantly, macrophages are elite producers of eicosanoids and other related lipid mediators during inflammation. Kupffer cells not only contribute to the production of inflammatory mediators they have also well-established connections to diet induced hepatic steatosis [28], PPAR-α activation, and non-genotoxic carcinogenesis [16]. From a metabolomics perspective, the role of macrophages in inflammatory signalling is mostly understood in the context of arachidonic acid metabolism. Arachidonic acid (AA; 20:4) is stored in PLs and during inflammation it is hydrolysed by cytosolic phospholipase A_2_ (cPLA_2_). It is also well established, that the ubiquitously expressed diacylglycerol kinases (DGKs) phosphorylate sn-1,2-DGs, with several DGKs exhibiting specificity for *sn*-1,2 DGs containing C20:4 [29, 30]. It is therefore reasonable to assume that a large proportion of PLs storing AA can be synthesised from DGs. On the other hand, precursors of phospholipase C (PLC), which cleaves off phosphoglycerol headgroups of PLs, exhibit a high abundance of C20:4 esterified at the sn-2 position of the glycerol backbone [29, 31].

**Fig. 7:**
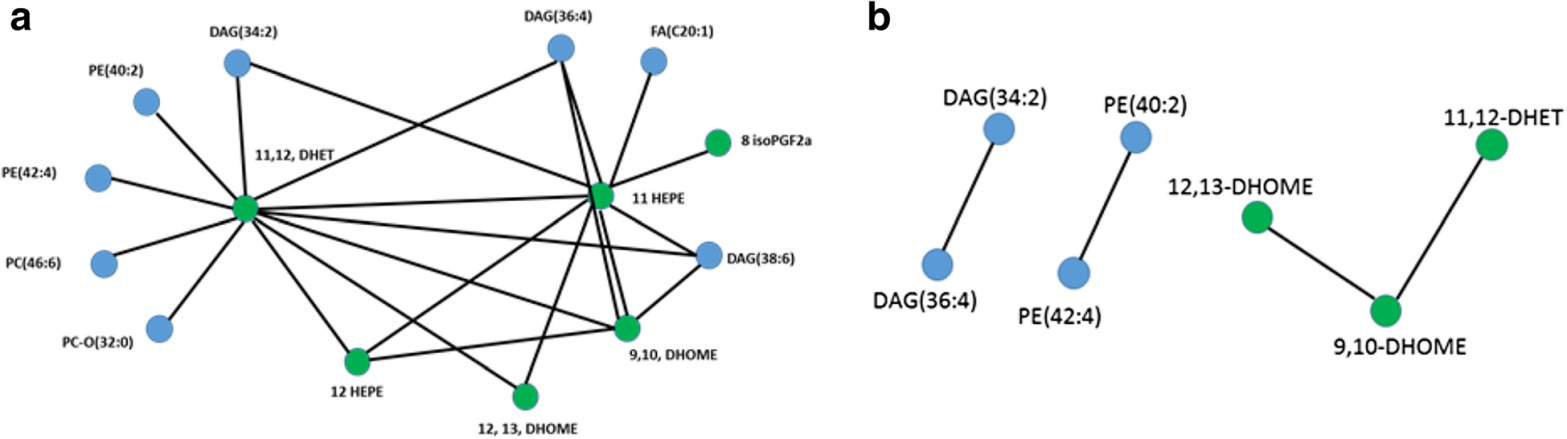
**a** A correlation network of the 14 selected variables based on a Pearson correlation coefficient of more than 0.8 (r > 0.8) linking with the relative liver weights. Lipid mediators (lipids both shorter and longer than eicosanoids) are shown in green whereas phospholipids are shown in blue. **b** Partial correlation with 14 variables are shown

In order to address the complexity and connections of lipid mediators with PLs and DGs, Pearson correlation and partial correlation analysis was applied using the eicosanoid and the DI-MS datasets for both positive and negative ionisation modes. There were 14 variables with a Pearson correlation ≥0.8, and all eicosanoids were found to positively correlate with the intact lipids (Fig. 7a, 7b). For those metabolites partial correlation analysis was performed and only subset of the metabolites found connected. This demonstrates that lipidomic profiling can offer insight about the source of downstream signalling effects in cells.

The central role of the liver in regulating systemic metabolism has put this organ in the spotlight for decades in toxicology, and yet many molecular mechanisms are still mostly unexplored. For example, AA metabolism as highlighted in the current study, and the metabolic concept, that the source of AA for eicosanoid production is based in PLs although widely accepted is being challenged [32]. Indeed, the pathways for AA liberation, how enzymes access their substrates, and how these mechanisms are controlled by PPARs remains essentially unresolved. We have demonstrated above the versatility of lipidomic data integration, and that the investigation of lipid signalling molecules is an important challenge for future research. In addition, our results highlight the need for further mechanistic studies to better understand how lipid remodelling at the gross level (e.g. DI-MS methods) produces changes in lipid signalling molecules such as eicosanoids.

## Conclusions

In this study, we present a powerful strategy for integrating multiple -omics data using a machine learning algorithm (RF) and selecting discriminatory metabolites for partial correlation network analysis. We used RF regression and classification for integrating metabolomics data sets. In terms of comparing RF to other similar approaches, Scott et al., 2013 [14] studied and tested 28 classifiers on NMR spectroscopy or MS data of different origins as the training sets for various multivariate tools. Data came from four metabolomics or food projects, whose class numbers differed. Random forests performed best on high-dimensional data, but was only used in 4.5 % of papers. This procedure can handle high dimensional data (for example, where the number of metabolites is much larger than the number of samples) and has an internal crossvalidation procedure (using the OOB samples). However, it could be considered a limitation that a RF model by default will use all variables simultaneously and if we want to perform variable selection, we need to set a threshold on the number of variables or we need to select variables based on a significance criterion or a variable selection procedure. The backward elimination procedure implemented in the package varSelRF uses the OOB as minimization criterion. Thus, the OOB performances achieved with the reduced model is likely to be biased; one possible solution would be applying the cross validation based protocol discussed in [33]. We used each of the predictor data separately (Fig. 3a) because we would like to interrogate each of the data sets independently to determine if those datasets are informative or not. If we would have combined the data into a single matrix, we would have missed that information. For example, aqueous open profiling data with all the variables gives rise to more than a 70 % class error. Thus, this data set is probably not useful as it is not predictive enough for a given response variable. This approach will help biologists if they want to perform targeted experiments in the future following an open profiling metabolomics experiment as part of the discovery phase. Thus, this data fusion strategy will help them to identify leads from a huge pool of –omics data sets. To the best of our knowledge, no such integrative approach have been utilised to link classical hepatic parameters with metabolomic and/or lipidomic datasets. We believe, that by linking classical toxicology parameters with metabolite markers, more accurate and early detection of toxicity can be facilitated. In addition, the presented approach can easily be applied in human to discover novel relationships in multi -omic data sets. In conclusion, our integrative approach offers a good starting point for addressing the complexity of interrelated metabolites, although more studies are needed for validation and to further explore the interrelation between metabolism, signalling, and disease.

## Additional files

Additional file 1: List of clinical chemistry analysis performed and their full descriptions. (DOCX 13 kb)
Additional file 2: Method descriptions for GC-MS, LC-MS/MS analysis of intact lipids, targeted analysis of aqueous metabolites and eicosanoids by LC-MS. (DOCX 20 kb)
Additional file 3: List of lipidomics, metabolomics and clinical chemistry data set. (XLSX 897 kb)
Additional file 4: The R- Scripts used in the analysis. (TXT 4 kb)
Additional file 5: Box plot of selected variables using RF classification. (PPTX 104 kb)
Additional file 6: Table of classical toxicology and variation explained (Q2) linking different data sets. (PPTX 60 kb)

## Abbreviations

AA: Arachidonic acid
AST: Aspartate transaminase
COX: Cyclooxygenase
cPLA2: Phospholipase A2
cPLA_2_: Phospholipase C
CYP: Cytochrome P450
DGKs: Diacylglycerol kinases
DGLA: Dihomo-γ-linolenic acid
DGs: Diglycerides
DHET: Dihydroxyeicosatrienoic acid
DHOME: Dihydroxyoctadecenoic acid
DI-MS: Direct infusion mass spectrometry
EI: Electron ionisation
EPA: Eicosapentaenoic acid
Etn: Ethanolamine
FAME: Fatty acid methyl esters
GC-MS: Gas-chromatography mass-spectrometry
HEPE: Hydroxyeicosapentaenoic acid
HETE: Hydroxyeicosatetraenoic acid
LA: Linoleic acid
LC-MS/MS: Liquid chromatography tandem mass spectrometry
LOX: Lipoxygenase
MetS: Metabolic syndrome
MS: Mass spectrometry
NRs: Nuclear hormone receptors
OOB: Out-of-bag
PC: Phosphocholine
PE: Phosphoethanolamine
PG: Phosphoglycerol
PLs: Phospholipids
PPAR: Peroxisome proliferator-activated receptors
RF: Random forest
SD: Sprague–Dawley
sEH: Soluble-epoxy-hydrolase
SITR1: Siturin 1
SM: Sphingomyelin
TG: Triacylglycerol or triglycerides
THET: Trihydroxyeicosatetraenoic acid
TriHOME: Trihydroxy-octadecenoic acid
