# Supplementary material for: Integration of metabolomics, lipidomics and clinical data using a machine learning method
Source: BMC Bioinformatics. 2016 Nov 22;17(Suppl 15):37–49. doi: 10.1186/s12859-016-1292-2 (PMC5133491; doi:10.1186/s12859-016-1292-2)
Supplement: Additional file 1: — List of clinical chemistry analysis performed and their full descriptions. (DOCX 13 kb) [file 12859_2016_1292_MOESM1_ESM.docx]

**Additional file 1**

**List of clinical chemistry analysis performed and their full descriptions**

| **Clinical chemistry** | **Full name** |
| --- | --- |
| Hb (g/dL) | Haemoglobin |
| RBC (mil/cmm) | Red blood cells |
| PCV (%) | Packed cell volume |
| RET ABS(mil/cmm) | Reticulocytes absolute |
| MCV(fL) | Mean cell volume |
| MCH (pg) | Mean cell haemoglobin |
| MCHC (g/dL) | Mean cell haemoglobin concentration |
| RDW (%) | Red cell distribution width |
| PLAT (1000/cmm) | Platelet count |
| PT (s) | Prothrombin time |
| APTT (s) | Activated partial thromboplastin time |
| WBC (1000/cmm) | White blood cell |
| N (1000/cmm) | Neutrophil |
| L (1000/cmm) | Lymphocytes |
| M (1000/cmm) | Monocytes |
| E (1000/cmm) | Eosinophil |
| B (1000/cmm) | B lymphocytes |
| AST (IU/L) | Aspartate amino transaminase |
| ALT (IU/L) | Alanine amino transaminase |
| ALK PHOS (IU/L) | Alkaline phosphatase |
| Na (mmol/L) | Sodium |
| K (mmol/L) | Potassium |
| Ca (mmol/L) | Calcium |
| IN PHOS(mmol/L) | Inorganic phosphorus |
| Cl (mmol/L) | Chlorine |
| T PROT(g/L) | Total protein |
| ALBUMIN (g/L) | Albumin |
| TOT CHOL (mmol/L) | Total Cholesterol |
| GLUC (mmol/L) | Glucose |
| UREA (mmol/L) | Urea |
| T BILI (umol/L) | Total Bilirubin |
| CREAT (umol/L) | Creatinine |
| TRIGS (umol/L) | Triglyceride |
| CPK (IU/L) | Creatine phosphokinase |
