# Supplementary material for: Integration of metabolomics, lipidomics and clinical data using a machine learning method
Source: BMC Bioinformatics. 2016 Nov 22;17(Suppl 15):37–49. doi: 10.1186/s12859-016-1292-2 (PMC5133491; doi:10.1186/s12859-016-1292-2)
Supplement: Additional file 2: — Method descriptions for GC-MS, LC-MS/MS analysis of intact lipids, targeted analysis of aqueous metabolites and eicosanoids by LC-MS. (DOCX 20 kb) [file 12859_2016_1292_MOESM2_ESM.docx]

**Additional file 2**

**GC-MS analysis of fatty acid methyl esters (FAMEs)**

For GC-MS, the resulting organic fractions from the chloroform: methanol extraction were used. Samples were reconstituted in 1 mL of methanol: chloroform 2:1 and a fifth of each sample (200µL) was transferred to a 3 mL glass vial. The 200 µL aliquots were dried under nitrogen before being derivatised with a methylating agent which forms fatty acid methyl esters (FAMEs) of carboxylic acids. Chloroform: methanol (1:1, 100 μl), boron trifluoride in methanol (10%, 125 μl) and 150 µL D-25-tridecanoic acid (200 µM in chloroform) were added to the dried extracts. Samples were vortex mixed and heated to 80˚C for 90 min. After cooling, 300 µL water and 600 µL hexane were added. The samples were vortex mixed, the lower aqueous layer was removed and the remaining organic layer dried under nitrogen. The samples were reconstituted in 150 μl hexane and transferred to autosampler vials prior to analysis using a Trace GC Ultra coupled to a DSQ II single-quadrupole mass spectrometer (Thermo Scientific, Hemel Hempstead, Hertfordshire). Samples were injected onto a Zebron™ ZB-WAX column (100% polyethylene glycol 30 m x 0.25 mm ID, 0.25 µm film thickness). The injector temperature was 230˚C and the flow rate of helium was 1.2 mL/min. The column was held at 60˚C for 2 min, after which the temperature was increased to 150 ˚C at a rate of 15˚C/min, and finally increased to 240 ˚C at a rate of 2.5˚C/min. The transfer line temperature was maintained at 240˚C, while the ion source was at 250˚C, operating at 70 eV for electron ionisation (EI). The detector was initiated after 240 s, and full scan spectra were collected over a range of 50-650 *m/z*.

**Open profiling LC-MS/MS analysis of intact lipids**

For LC-MS/MS analysis of lipids, the organic fractions of the stock chloroform: methanol extraction were used. A 10 µL aliquot, comprising one hundredth of the organic fraction, was diluted into 90 µL of methanol-chloroform (2:1) containing 20 µM 1,2-diheptadecanoyl-sn-glycero-3-phosphocholine (PC (17:0/17:0)) (Avanti Polar Lipids Inc., Alabaster, Alabama, US) The instrumentation comprised a Xevo G2 Quadrupole Time of Flight (QToF) mass spectrometer with a Z-spray electrospray source (Waters Ltd., Elstree, Hertfordshire, UK) coupled to an ACQUITY Ultra Performance Liquid Chromatography (UPLC) system (Waters Ltd., Elstree, Hertfordshire, UK). Separation of species was achieved using an Acquity CSH C18, 1.7 µm (2.1 x 100 mm) column (Waters Ltd., Elstree, Hertfordshire, UK). Mobile phase A consisted of 10 mM ammonium formate in acetonitrile: water (6:4), whilst mobile phase B contained 10 mM ammonium formate in isopropanol: acetonitrile (9:1). The concentration of mobile phase B was increased from 40-100% over 18 min, then equilibrated to 40% B for 2 min at a flow rate of 0.4 mL/min. The electrospray source was operated in positive ion mode with the source temperature set at 80 °C and a cone gas flow of 100 L/h. The desolvation gas temperature was 250 °C and the nebuliser gas flow rate was set at 700 L/h. The capillary voltage was 3 kV and the cone voltage 50 V. Mass spectrometric data were collected from 50-1200 *m/z* in profiling scan mode.

**Targeted analysis of aqueous metabolites**

For LC-MS/MS analysis aqueous phase metabolites resulting from the chloroform: methanol extractions were used. The entire fraction was dissolved in 300 µl of 70:30 acetonitrile: water containing 20 µM universally ^13^C- and ^15^N- labelled glutamate. Samples were vortex mixed, sonicated, centrifuged, (17,000 x *g*, 5 min) pipetted into auto sampler vials and analysed using an AB Sciex 5500 Qtrap mass spectrometer (AB Sciex UK Limited, Warrington, Cheshire) coupled to a SIL20-A LC system (Shimadzu Corp., Kyoto, Japan). Mobile phase A consisted of 100 mM ammonium acetate, mobile phase B was acetonitrile, and the flow rate was 0.3 mL/min. Two microliters of each sample was injected, and analytes separated using a 100 mm ZIC-HILIC column with 2.1 mm ID and 3.5 µm particle size (Sequant, Umeå, Sweden). A linear gradient was used, starting at 20 % A for 2 min, followed by an increase to 50 % A over 10 min, and finally a 3 min re-equilibration. Metabolites of interest were measured in positive ionisation mode with unscheduled multiple reaction monitoring events (MRMs), using a source temperature of 500 ºC, an ion spray voltage of 4.5 kV and a dwell time of 50 ms. Peaks were integrated by the Quantitation Wizard within Analyst™ version 1.6 by AB Sciex Ltd. (Warrington, Cheshire, UK) and normalised against wet tissue weight and to the intensity of the internal standards.

**Analysis of acyl-carnitines**

Acyl-carnitines were measured according to the method described by Roberts *et al*. [13]. Briefly, 100 µL internal standard solution mix (1.63 µM [D9] free carnitine, 0.3 µM [D3] acetyl carnitine, 0.06 µM [D3] propionyl-carnitine, 0.06 µM [D3] butyryl-carnitine, 0.06 µM [D9] isovarelyl-carnitine, 0.06 µM [D3] octanoyl-carnitine, 0.06 µM [D9] myristoyl-carnitine, and 0.12 µM [D3] palmitoyl-carnitine, Cambridge Isotope Laboratories, Andover, MA, USA) was added to 40 µL of the organic fraction of the methanol: chloroform extraction and the resulting mixture were dried down under nitrogen and derivatised with 100 µL of 3 M butanolic-HCl (Sigma-Aldrich, Louis, Missouri, USA). Samples were evaporated under nitrogen, re-constituted and sonicated in 4:1 acetonitrile: 0.1% formic acid in water before transferring them to autosampler vials. Samples were analysed using an AB Sciex 5500 QTRAP mass spectrometer (AB Sciex UK Limited, Warrington, Cheshire) coupled to an Acquity UPLC system. Mobile phase A consisted of 0.1 % formic acid in water, while mobile phase B was acetonitrile. Two microliters of each sample was injected onto a Synergi Polar RP phenyl ether column (100 mm × 2.1 mm, 2.5 µm) supplied by Phenomenex (Macclesfield, Cheshire, UK). The analytical gradient started at 30 % B, followed by a linear increase to 100 % B over 3 min. The gradient was then held at 100 % B for 5 min, after which it was returned to the re-equilibration level of 30 % B for 2 min. A flow rate of 0.5 mL/min was used throughout. Data were analysed using the Quantitation Wizard within Analyst™ version 1.6 by AB Sciex Ltd. (Warrington, Cheshire, UK) and normalised against wet tissue weight and to the intensity of the internal standard.

#### Targeted analysis of eicosanoids by LC-MS

Analysis was performed using a 4000 QTRAP triple quadrupole mass spectrometer (AB Sciex UK Limited, Warrington, Cheshire) coupled to an Acquity ultra performance liquid chromatography (UPLC) system (Waters Corp., Milford, MA). The autosampler was maintained at 4 ˚C, LC separation was achieved using a Luna, 3 μm particle size, 150 × 2 mm column (Phenomenex Macclesfield, Cheshire, UK). The gradient of mobile phase A (0.1% acetic acid in water) and B (0.1% acetic acid in acetonitrile: methanol 80:20). The flow rate was held at 0.4 mL/min. Metabolites of interest were measured in negative ionisation mode with unscheduled multiple reaction monitoring events (MRMs) .Peaks were integrated by the Quantitation Wizard within Analyst™ version 1.6 by AB Sciex Ltd. (Warrington, Cheshire, UK) and normalised against wet tissue weight and to the intensity of the internal standard
