# Supplementary figures and images for: Integration of metabolomics, lipidomics and clinical data using a machine learning method
Source: BMC Bioinformatics. 2016 Nov 22;17(Suppl 15):37–49. doi: 10.1186/s12859-016-1292-2 (PMC5133491; doi:10.1186/s12859-016-1292-2)

## Slide 1
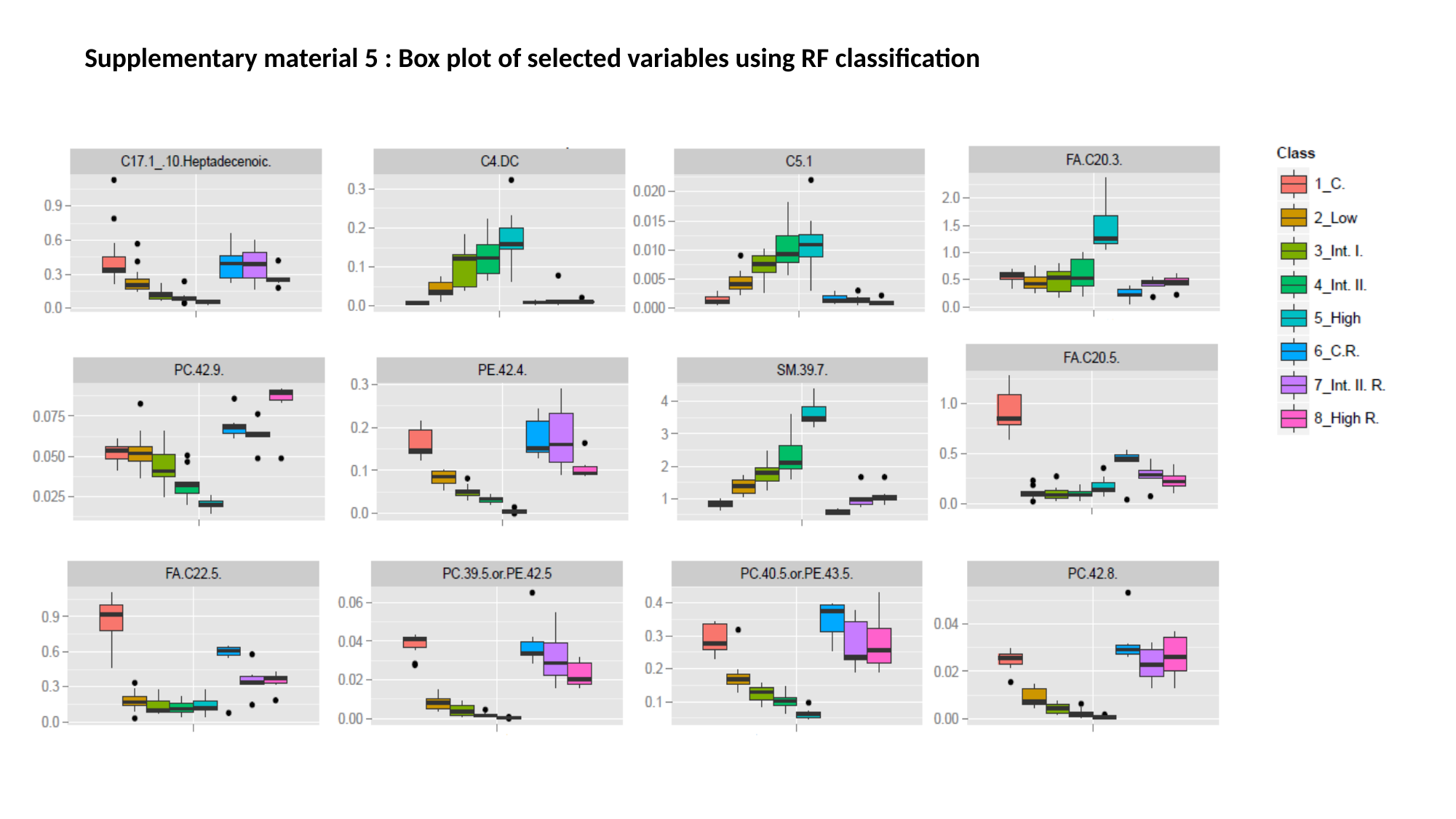

Supplementary material 5 : Box plot of selected variables using RF classification

Supplement: Additional file 5: — Box plot of selected variables using RF classification. (PPTX 104 kb) [file 12859_2016_1292_MOESM5_ESM.pptx]
