# Supplementary material for: Integration of metabolomics, lipidomics and clinical data using a machine learning method
Source: BMC Bioinformatics. 2016 Nov 22;17(Suppl 15):37–49. doi: 10.1186/s12859-016-1292-2 (PMC5133491; doi:10.1186/s12859-016-1292-2)
Supplement: Additional file 6: — Table of classical toxicology and variation explained (Q2) linking different data sets. (PPTX 60 kb) [file 12859_2016_1292_MOESM6_ESM.pptx]

## Slide 1
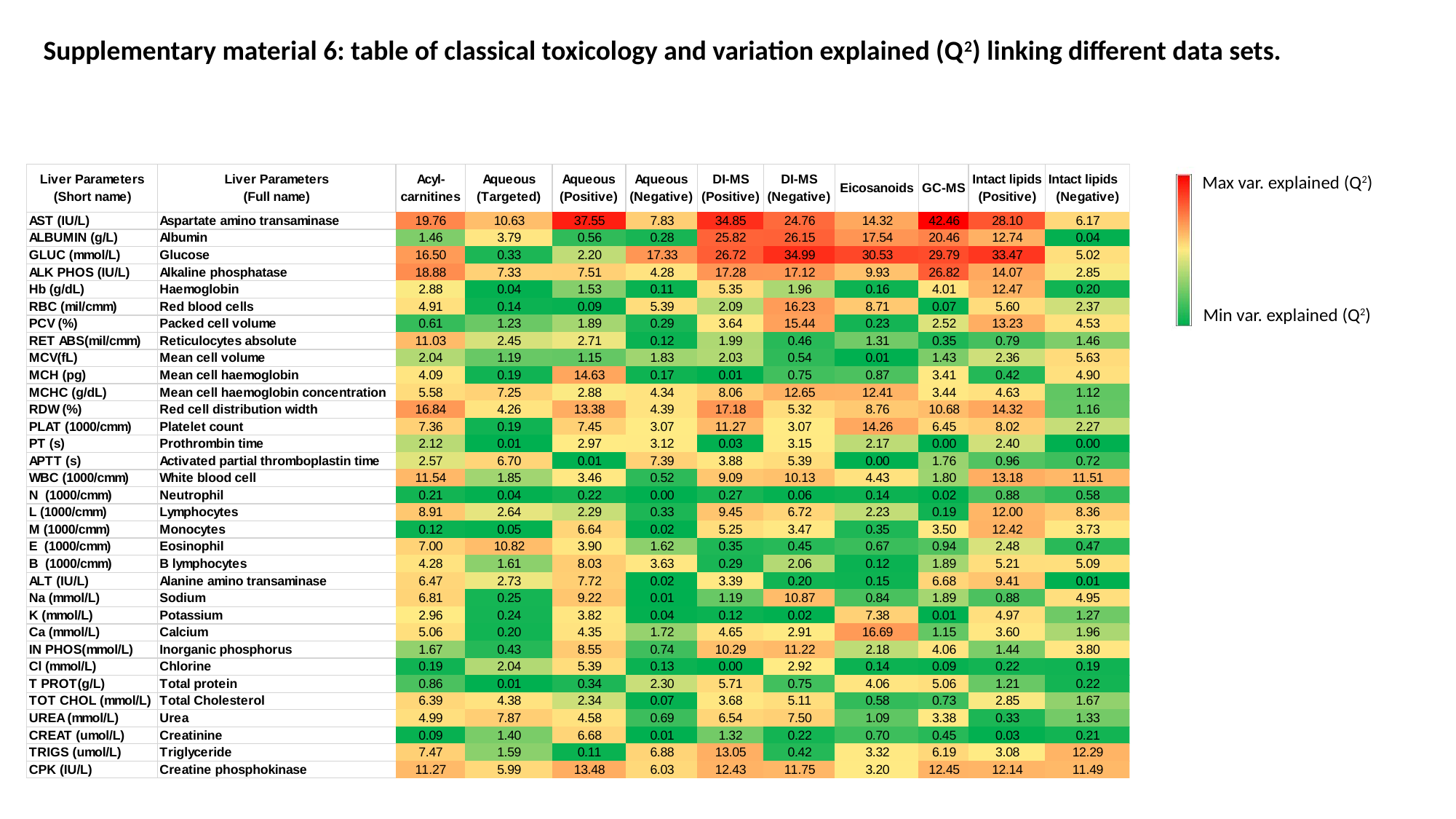

Supplementary material 6: table of classical toxicology and variation explained (Q2) linking different data sets.
Max var. explained (Q2)
Min var. explained (Q2)
